# Low levels of circulating anti-ageing hormone Klotho predict the onset and progression of diabetic retinopathy

**DOI:** 10.1177/1479164120970901

**Published:** 2020-11-21

**Authors:** Antonella Corcillo, Nikolaos Fountoulakis, Angela Sohal, Frederick Farrow, Salma Ayis, Janaka Karalliedde

**Affiliations:** 1 School of Cardiovascular Medicine & Sciences, King’s College London, London, UK; 2 Guy’s and St Thomas NHS Foundation Trust, Guy’s Hospital, London UK

**Keywords:** Type 2 diabetes, Klotho, retinopathy, biomarker, anti-ageing

## Abstract

**Background:**

Klotho is a circulating anti-ageing hormone that predicts progression of cardiovascular and renal disease. The role of Klotho in diabetic retinopathy is unknown.

**Methods:**

We performed a single-centre observational study of 81 people (males 62%) with type 2 diabetes followed for a median of 44 months. Circulating levels of Klotho and other markers, were measured from stored samples. The primary outcome was progression of retinopathy defined as new onset retinopathy or step change in retinopathy grading.

**Results:**

During follow-up, 46 (57%) people reached the primary outcome. People with progression of retinopathy had lower levels of serum Klotho as compared to those without (median (interquartile range) 226.9 (171.1–394.0) vs 484.5 (221.8–709.9) pg/ml, *p* = 0.001). In multivariable logistic regression analyses, baseline Klotho level was the only variable independently associated with reduced risk of progression of retinopathy. Our results suggest that a halving of circulating Klotho levels increases the risk of retinopathy progression by 44%.

**Conclusion:**

In people with type 2 diabetes, lower circulating levels of the vascular protective hormone Klotho are associated with increased risk of progression of diabetic retinopathy. Klotho may be a novel biomarker and potential treatment target for diabetic eye disease.

## Introduction

Klotho is a circulating anti-ageing hormone with anti-oxidative and anti-inflammatory properties that has demonstrated vascular protective effects in animal studies.^1^ Recent data suggests that low levels of circulating Klotho predicts increased risk of progression of cardiovascular and renal disease.^2^ The role of Klotho in predicting progression of diabetic retinopathy has not been studied and is currently unknown.

## Material and methods

We performed a single-centre observational follow-up study of 81 patients with type 2 diabetes followed for a median of 44 (interquartile range (IQR) 21.5–62.0) months. The primary outcome was progression of retinopathy defined as new onset retinopathy or step change in retinopathy grading. In view of the lack of previous studies in this area and to generate future sample size calculations, we chose this pragmatic composite endpoint for our proof of concept study.

Retinopathy was evaluated by monoscopic fundus photos of dilated pupils using a nonmydriatic camera. Digital retinal images were graded according to the United Kingdom National Health Service Diabetic Eye Screening Programme. Serum levels of Klotho, phosphorus, calcium, vitamin D and fibroblast growth factor-23 (FGF-23) levels were measured from stored samples collected at baseline as described previously.^3^ None of the patients were on vitamin D, calcium or phosphate supplements at baseline. All patients had an estimated glomerular filtration (eGFR) rate of >30 ml/min by Modification of Diet in Renal Disease (MDRD) Study equation. Clinical, biochemical and anthropometric data were collected from electronic health records.

Descriptive statistics were used for the analysis of demographic and clinical features of the cohort. Klotho levels were log transformed before calculation because of their positively skewed distribution. Between-group differences were compared by unpaired *t* test (for continuous parametrically distributed variables) and Mann-Whitney test (for continuous non-parametrically distributed variables). A χ^2^ test was used to compare categorical variables between groups. Multivariate logistic regression analysis was performed to evaluate the variables significantly associated with progression of retinopathy. Data are given as mean ± SD, percentage for categorical variables, or median and IQR for variables not normally distributed. A two-tailed *p* value <0.05 was considered significant. Statistical analyses were performed with SPSS version 25.0 (SPSS, Chicago, IL, USA).

## Results

The mean age (range) of our cohort was 61 (48–78) years with a median diabetes duration of 11.8 years. Patients were predominantly male (62%) and 91% were on renin angiotensin aldosterone system (RAAS) blockade. Baseline median (IQR) circulating serum Klotho level was 265 (184.7–567.78) pg/ml.

At baseline, 47 (58%) patients had evidence of diabetic retinopathy. Of these 47 patients with baseline retinopathy, the majority (80%) had background retinopathy, with the remainder having pre-proliferative (10%) or proliferative (10%) retinopathy respectively.

During the follow up observation period, 46 (57%) patients reached the primary endpoint of progression of retinopathy.

Table 1 details selected clinical and biochemical variables between patients with retinopathy progression as compared to those without progression. Patients with progression of retinopathy as compared to those without were similar in clinical features including HbA1c, serum phosphate, FGF-23, vitamin D levels, demographics and traditional risk factors for retinopathy (Table 1). However, we observed the novel finding of lower levels of circulating Klotho (median (IQR) 226.9 (171.1–394.0) vs 484.5 (221.8–709.9) pg/ml, *p* = 0.001) in patients with progression of retinopathy as compared to those without. The only other parameters that were significantly different between the two groups were serum calcium and eGFR (Table 1). There were no significant differences observed in biomarkers and markers of vascular inflammation and oxidative stress associated with retinopathy such as oxidized low-density lipoprotein (ox-LDL), high sensitivity C reactive protein (hsCRP) and vascular cell adhesion molecule (VCAM-1) between the two groups (results were available for sub-group of 54 patients). Neither duration of follow-up nor severity of retinopathy at baseline were determinant factors for progression of retinopathy.

**Table 1.** Selected baseline clinical and biochemical variables in a cohort of 81 patients with diabetes with and without progression of diabetic retinopathy.

	No progression of retinopathy (*n* = 35)	Retinopathy progression (*n* = 46)	*p* value
Age, years	60.7 ± 9.3	61.2 ± 8.8	0.79
Duration of diabetes, years	13.8 ± 8.2	12.7 ± 11.1	0.62
RAAS blockade	89%	93%	0.44
BMI, kg/m^2^	32.3 ± 6.0	32.6 ± 6.4	0.81
HbA1c, % (mmol/mol)	7.7 ± 1.4 (61 ± 7)	7.6 ± 1.4 (60 ± 7)	0.67
SBP, mmHg	151.6 ± 16.5	156.7 ± 13.9	0.13
DBP, mmHg	79.6 ± 10.0	81.5 ± 9.6	0.40
eGFR, ml/min/1.73 m^2^/year	69.0 ± 27.3	81.7 ± 23.7	0.03
Total cholesterol, mmol/l	4.3 ± 1.0	4.3 ± 1.1	0.94
Klotho, pg/ml*	484.5 (221.8–709.9)	226.9 (171.1–394.0)	0.001
FGF-23, RU/ml	56.5 ± 42.4	58.9 ± 32.2	0.81
Serum calcium, mg/l	2.3 ± 0.1	2.4 ± 0.1	0.02
Phosphate, mmol/l	1.1 ± 0.2	1.1 ± 0.4	0.45
25 vitamin D, ng/ml	44.8 ± 20.3	35.9 ± 21.6	0.12
hsCRP, mg/l**	4.7 ± 5.1	4.0 ± 4.7	0.61
VCAM-1, ng/ml**	508.3 ± 204.2	606.9 ± 276.3	0.36
Ox-LDL, U/l**	48.9 ± 22.0	48.2 ± 20.6	0.61

BMI: body mass index; eGFR: estimated glomerular filtration rate; SBP: systolic blood pressure; DBP: diastolic blood pressure; RAAS: renin angiotensin aldosterone system; FGF-23: fibroblast growth factor 23; hsCRP: high sensitivity C reactive protein; VCAM-1: vascular cellular adhesion molecule-1; Ox-LDL: oxidized low-density lipoprotein.

Mean ± SD, *n* (%) or * median (interquartile range).

** Results available for sub-group of 54 patients (*n* = 17 without progression and *n* = 37 with progression of retinopathy).

The number of patients who developed the individual components of the composite retinopathy endpoint were 24 for new onset retinopathy and 22 for progression of retinopathy respectively. In post-hoc analyses, we did not observe a significant effect of soluble Klotho levels on these individual components of the primary composite endpoint. Of the cohort of 81 patients, 22% (*n* = 18) had diabetic maculopathy at baseline and we did not observe any significant association between Klotho levels and progression of maculopathy.

In multivariable logistic regression analyses, baseline log Klotho levels was the only variable independently associated with reduced risk of progression of retinopathy (OR 0.067, 95% CI 0.006–0.693, *p* = 0.015). Serum calcium and eGFR were not significantly associated with retinopathy progression. Our results suggest that a halving of circulating Klotho levels increases the risk of retinopathy progression by 44%. The significant effect of Klotho was also independent of traditional risk factors for retinopathy progression (e.g. systolic and diastolic blood pressure measures, HbA1c, duration of diabetes) in multivariable analyses.

## Discussion

There is limited information on Klotho and diabetic retinopathy in the literature. In one recent cross-sectional study by Ji et al.^4^ in 60 patients with diabetes (of whom 33 had diabetic retinopathy), the authors observed that more advanced stages of diabetic eye disease were associated with lower circulating Klotho levels and that Klotho pre-treatment ameliorated palmitic acid induced apoptosis in human retinal endothelial cells. Limitations of the study was the inclusion patients with advanced renal disease, lack of information on concomitant medication (such as RAAS) which influence klotho levels and absence of prospective data.

In a study by Słomiński et al.,^5^ Klotho gene polymorphism were observed to protect against the development of retinopathy in patients with type 1 diabetes and the polymorphism was associated with lower levels of inflammatory markers, pro-angiogenic factors and adhesion molecules. The authors hypothesised that their results may be related to increased Klotho protein levels or activity of the protein which could protects against microvascular inflammation and endothelial dysfunction that drive onset and progression of retinopathy.

The strengths of our study include the use of standardised and robust diabetic eye screening data from digital records with the long term follow up in a well characterised cohort of type 2 diabetes. We also measured several emerging markers of retinopathy risk such as VCAM-1, ox-LDL, hsCRP in a sub-group of patients and other hormones associated with Klotho such as FGF-23 and vitamin D which were not significantly different between groups. HbA1c was similar in the two groups. We excluded patients with advanced kidney disease and those on treatment with vitamin D, calcium or phosphate supplements or binders as this may influence Klotho and FGF23 levels and could confound interpretation of the results. The limitations of our study are the relatively small sample size, the single centre design and the lack of additional measures of angiogenic markers/inflammatory mediators in the full cohort. The primary retinopathy endpoint of our study was a composite of new onset retinopathy or step change in retinopathy grading. A further limitation of our study was that, possibly due to the small sample size, we were unable determine the effects of Klotho on these individual components. Further larger studies are required to determine the effect of Klotho levels on these distinct retinopathy endpoints. Our observations and results establish the rationale for future work in this important area. Our study was also not designed to explain the potential pathophysiology between circulating Klotho level perturbations and progression of retinopathy. We speculate that the well-established anti-inflammatory, anti-oxidative and anti-apoptotic vascular effects of Klotho may also protect against diabetic endothelial dysfunction and microvascular damage that characterise diabetic retinopathy, however further mechanistic studies are required to explain our findings.

In conclusion, in our cohort of people with type 2 diabetes, lower circulating levels of the vascular protective hormone Klotho are associated with increased risk of new onset and progression of diabetic eye disease. Klotho may be a novel biomarker of diabetic retinopathy and a potential treatment target for diabetic eye disease.
